# Resveratrol Prevents High Fluence Red Light-Emitting Diode Reactive Oxygen Species-Mediated Photoinhibition of Human Skin Fibroblast Migration

**DOI:** 10.1371/journal.pone.0140628

**Published:** 2015-10-21

**Authors:** Andrew Mamalis, Eugene Koo, R. Rivkah Isseroff, William Murphy, Jared Jagdeo

**Affiliations:** 1 Department of Dermatology, University of California Davis, Sacramento, CA, United States of America; 2 Dermatology Service, Sacramento VA Medical Center, Mather, CA, United States of America; 3 Department of Internal Medicine, University of California Davis, Sacramento, CA, United States of America; 4 Department of Dermatology, SUNY Downstate Medical Center, Brooklyn, NY, United States of America; Massachusetts General Hospital, UNITED STATES

## Abstract

**Background:**

Skin fibrosis is a significant medical problem that leads to a functional, aesthetic, and psychosocial impact on quality-of-life. Light-emitting diode-generated 633-nm red light (LED-RL) is part of the visible light spectrum that is not known to cause DNA damage and is considered a safe, non-invasive, inexpensive, and portable potential alternative to ultraviolet phototherapy that may change the treatment paradigm of fibrotic skin disease.

**Objective:**

The goal of our study was to investigate the how reactive oxygen species (ROS) free radicals generated by high fluence LED-RL inhibit the migration of skin fibroblasts, the main cell type involved in skin fibrosis. Fibroblast migration speed is increased in skin fibrosis, and we studied cellular migration speed of cultured human skin fibroblasts as a surrogate measure of high fluence LED-RL effect on fibroblast function. To ascertain the inhibitory role of LED-RL generated ROS on migration speed, we hypothesized that resveratrol, a potent antioxidant, could prevent the photoinhibitory effects of high fluence LED-RL on fibroblast migration speed.

**Methods:**

High fluence LED-RL generated ROS were measured by flow cytometry analysis using dihydrorhodamine (DHR). For purposes of comparison, we assessed the effects of ROS generated by hydrogen peroxide (H_2_O_2_) on fibroblast migration speed and the ability of resveratrol, a well known antioxidant, to prevent LED-RL and H_2_O_2_ generated ROS-associated changes in fibroblast migration speed. To determine whether resveratrol could prevent the high fluence LED-RL ROS-mediated photoinhibition of human skin fibroblast migration, treated cells were incubated with resveratrol at concentrations of 0.0001% and 0.001% for 24 hours, irradiated with high fluences LED-RL of 480, 640, and 800 J/cm^2^.

**Results:**

High fluence LED-RL increases intracellular fibroblast ROS and decreases fibroblast migration speed. LED-RL at 480, 640 and 800 J/cm^2^ increased ROS levels to 132.8%, 151.0%, and 158.4% relative to matched controls, respectively. These LED-RL associated increases in ROS were prevented by pretreating cells with 0.0001% or 0.001% resveratrol. Next, we quantified the effect of hydrogen peroxide (H_2_O_2_)-associated ROS on fibroblast migration speed, and found that while H_2_O_2_–associated ROS significantly decreased relative fibroblast migration speed, pretreatment with 0.0001% or 0.001% resveratrol significantly prevented the decreases in migration speed. Furthermore, we found that LED-RL at 480, 640 and 800 J/cm^2^ decreased fibroblast migration speed to 83.0%, 74.4%, and 68.6% relative to matched controls, respectively. We hypothesized that these decreases in fibroblast migration speed were due to associated increases in ROS generation. Pretreatment with 0.0001% and 0.001% resveratrol prevented the LED-RL associated decreases in migration speed.

**Conclusion:**

High fluence LED-RL increases ROS and is associated with decreased fibroblast migration speed. We provide mechanistic support that the decreased migration speed associated with high fluence LED-RL is mediated by ROS, by demonstrating that resveratrol prevents high fluence LED-RL associated migration speed change. These data lend support to an increasing scientific body of evidence that high fluence LED-RL has anti-fibrotic properties. We hypothesize that our findings may result in a greater understanding of the fundamental mechanisms underlying visible light interaction with skin and we anticipate clinicians and other researchers may utilize these pathways for patient benefit.

## Introduction

Skin fibrosis is a significant medical problem that has a functional, aesthetic, and psychosocial impact on a patient’s quality-of-life.[1] Skin fibrosis results from altered cellular pathways initiated by chronic injury or inflammation. The unifying characteristics of skin fibrosis are increased fibroblast proliferation, collagen production, and transforming growth factor beta (TGF-Beta) signaling.[2] Skin fibrosis-derived fibroblasts also demonstrate increased migration speed compared to fibroblasts derived from normal human skin.[3,4] Unfortunately, there are few effective therapies for skin fibrosis and further research in this area is needed.

Ultraviolet (UV) phototherapy is currently used to treat some fibrotic skin diseases, however, UV light is associated with DNA damage leading to skin cancer and premature aging.[5,6] In contrast, light-emitting diode-generated 633-nm red light (LED-RL) is part of the visible spectrum that is not known to cause DNA damage and is considered a safe, non-invasive, inexpensive, and portable potential alternative to ultraviolet phototherapy that may change the treatment paradigm of fibrotic skin disease.[7–11] However, LED-RL’s molecular effects and underlying mechanisms are not well characterized. Low fluence LED-RL is associated with stimulatory effects such as wound healing and hair regrowth.[12] On the other hand, inhibitory effects are observed at higher fluences. For example, early clinical observations have demonstrated the anti-fibrotic effects of high fluence LED-RL, although the mechanisms underpinning this inhibition have remained largely unexplored.[13] The molecular effects of LED-RL are hypothesized to be modulated by mitochondrial cytochrome C oxidase.[14] The photoinhibitory effects of high fluence LED-RL lead to downstream modulation of key cellular processes involved in skin fibrosis.[11]

Resveratrol, a natural molecule derived from fresh grape skin, red wine, and Japanese knotweed, has significant antioxidant properities.[15,16] Research demonstrates that resveratrol is a potent antioxidant in human skin fibroblasts in vitro, and also modulates a number of key cellular pathways related to skin fibrosis including the TGF-beta/SMAD pathway, collagen deposition, and cellular proliferation.[17–20] These biologic effects in fibroblasts are hypothesized to be due to antioxidant capabilities, however, resveratrol can also cause direct effects on cellular pathways through other less-characterized mechanisms.[21] This makes resveratrol a good candidate for exploring the LED-RL photobiomodulatory effects on human skin fibroblasts.

The goal of our study was to investigate the mechanism by which reactive oxygen species (ROS) free radicals generated by high fluence LED-RL inhibit migration in skin fibroblasts. Fibroblast migration speed is increased in skin fibrosis, and we studied cellular migration speed in cultured human skin fibroblast model as a surrogate measure of high fluence LED-RL effect on fibroblast function. To ascertain the inhibitory role of ROS on migration speed, we hypothesized that resveratrol, a potent antioxidant scavenger of ROS, could prevent the photoinhibitory effects of high fluence LED-RL on fibroblast migration speed.

## Methods

### Cell Culture of Skin Fibroblasts

Monolayers of commercially available patient-derived primary human skin fibroblasts (AG13145, Coriell Institute) were cultured as previously described.[9] Fibroblasts were seeded in 35-mm tissue culture dishes at a density of 2x10^4^ cells per tissue culture dish. To demonstrate that resveratrol could modulate the effects of LED-RL, treated plates were incubated with resveratrol (Sigma-Aldrich, St. Louis, MO. USA) dissolved in DMEM at concentrations of 0.0001% and 0.001% for 24 hours prior to irradiation. Resveratrol concentrations studied were selected based upon previous demonstration of resveratrol’s antioxidant properties in human skin fibroblasts by our group.[16]

### High Fluence LED-RL Irradiation of Skin Fibroblasts

Fibroblasts were irradiated 24 hours after plating, using a 633-nm LED-RL array (Omnilux New-U, Photo Therapeutics, Carlsbad, CA) at a power density of 872.6 W/m2 and fluences of 480, 640, and 800 J/cm2 (1:31:41, 02:02:14, and 2:32:47 hr:min:sec, respectively) at room temperature. Each experimental plate receiving LED-RL treatment was compared with a temperature matched control plate to ensure that the measured effect was a result of LED treatment and not due to ambient light or environment. Controls were derived from the same stock of trypsinized cell suspension and plated to adhere in the same manner as the experimental conditions, taken out of the incubator at the same time as their matched treatment pairs, protected from the LED light source, and placed on a digital warming blocking with a negative feedback temperature control system set at 32°C. In all groups, medium temperatures throughout irradiation remained 32–34°C as measured using a digital thermometer (HH506RA, Omega Engineering, Stamford, CT). By remaining within physiologic temperature range, heat stress was not induced. LED-RL fluences were selected based upon previously published studies that demonstrated LED photoinhibition of skin fibroblast proliferation.[7,9,10]

### Hydrogen Peroxide Treatment of Skin Fibroblasts

Fibroblasts were treated with 1.2 mM hydrogen peroxide (H_2_O_2_, Sigma-Aldrich) 24 hours after plating for a duration of 30 minutes. Following treatment with H_2_O_2_, cells were washed with PBS and fresh medium was added. Concentration and duration of H_2_O_2_ exposure was selected based upon previous antioxidant studies by our group.[16,22,23]

### Flow-Cytometry Based Quantification of High Fluence LED-RL Generated Reactive Oxygen Species using Dihydrorhodamine

Intracellular ROS generation in fibroblasts was assessed as previously described.[16] Briefly, 2x10^4^ cells were plated per dish, intravitally stained with DHR, and then irradiated as described above. Following irradiation, cells were washed with PBS, collected with trypsin, and processed for flow cytometry analysis. The level of intracellular ROS was evaluated by flow cytometry analysis using a Guava easyCyte HT system at 525 nm. Mean fluorescent intensity was measured for each group and statistical analysis of data was performed using the paired two-tailed Student’s t -test, with significance level set at p < 0.05. In ROS experiments, the sample size was n = 3 for each experimental condition. Experiments were repeated twice to ensure reproducibility.

### Time-Lapse Video Single Cell Migration Microscopy

Time-lapse video migration experiments were performed as described with modifications on a Nikon TE-2000 microscope with a motorized stage.[24] For migration experiments, cultured human fibroblast cells were plated on 35 mm^2^ culture dishes with CO_2_-independent medium (Invitrogen) and incubated for 24 hours. In some experimental conditions, human fibroblasts were pretreated with resveratrol as described above. Cells were irradiated or treated with H_2_O_2_ as described above. Following irradiation, time-lapse images were captured every 30 minutes for 4 hours in an environmental chamber monitored under microscopy with the temperature maintained at 37°C. The time-lapse videos were generated using Volocity Image Software (PerkinElmer) and were analyzed using Openlab Software (PerkinElmer) to measure cell migration speed. “Migration speed” is the average speed in μm per minute that the cells travel in a 4-hour period. Statistical analysis of data was performed using the paired two-tailed Student’s t-test, significance level was set at p < 0.05. For each group, at least 40 cells were tracked to quantify migration speed and experiments were repeated twice to ensure reproducibility.

### Statistical Analysis

Statistical analyses with Student’s t-test was performed using GraphPad Prism version 6.00 for OSX (GraphPad Software, San Diego, CA, USA) to compare individual treatment arms and matched controls. The significance level was set at p < 0.05. Experimental design and sample size was chosen based upon guidance by a biostatistician.

## Results

**Fig 1:**
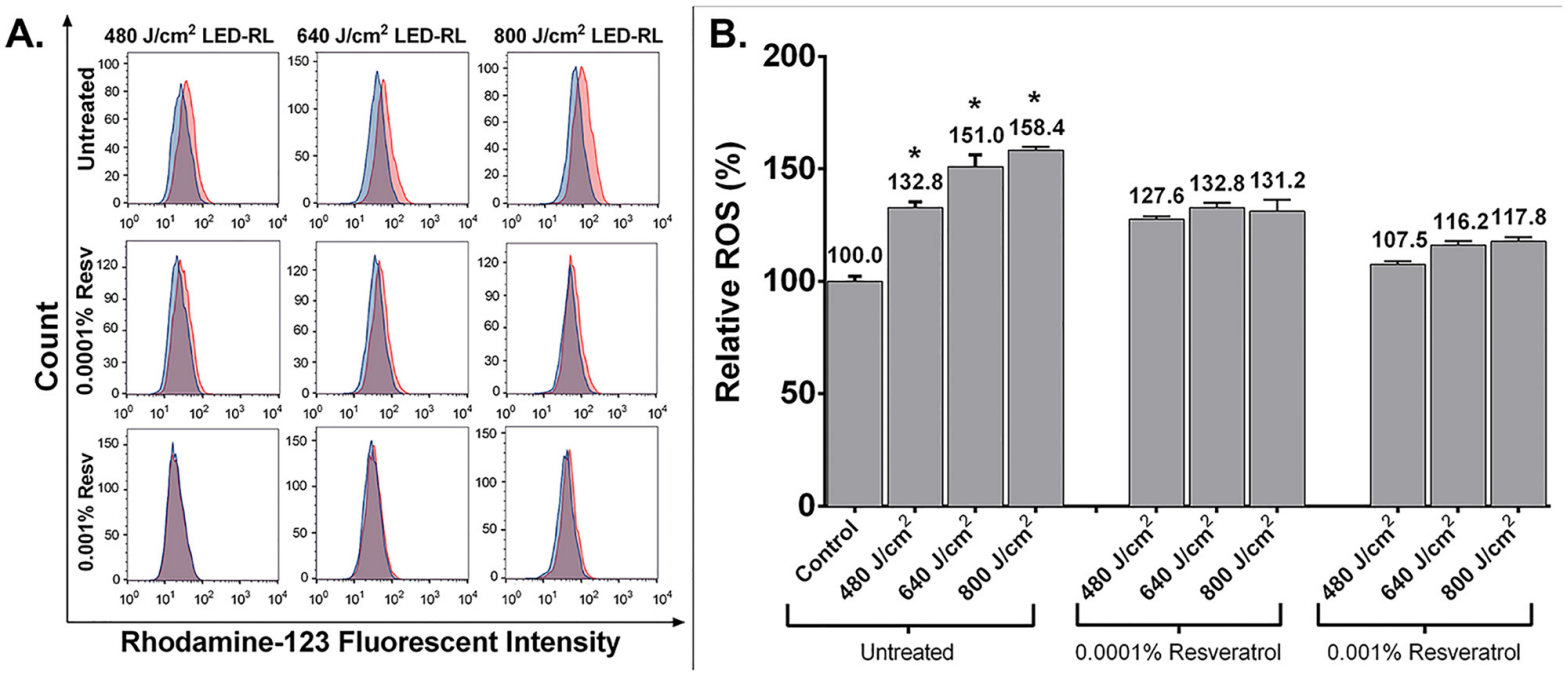
Light-emitting diode generated red light (LED-RL) at a fluences of 480, 640, and 800 J/cm^2^ significantly increased reactive oxygen species as measured by DHR. These increases in ROS were significantly prevented by pretreating cells with 0.0001% or 0.001% resveratrol. **(A)** Representative flow cytometry analysis of untreated and resveratrol pretreated fibroblasts stained with dihydrorhodamine. Red curves represent cells treated with LED-RL at the labeled fluence. Blue curves represent matched controls. **(B)** ROS levels are presented as median fluorescent intensity of DHR of treated cells relative to matched controls. ROS generation of 480, 640, and 800 J/cm^2^ LED-RL was 132.8±2.5% (p = 0.0006), 151.0±5.2% (p = 0.0001), 158.4±1.3% (p = 0.00184), respectively, compared to 100.0±2.2% in control. Pretreatment with 0.0001% resveratrol significantly reduced ROS generation of 480, 640, and 800 J/cm^2^ LED-RL to 127.6±1.3 (p = 0.000483), 132.8±2.1 (p = 0.00481), and 131.2±5.1 (p = 0.00022), respectively (p-values compared to matched LED-RL group that was not treated with resveratrol). Pretreatment with 0.001% resveratrol significantly reduced ROS generation of 480, 640, and 800 J/cm^2^ LED-RL to 107.5±1.4 (p = 2.3E-05), 116.2±1.7 (p = 0.00018), and 117.8±1.9 (p = 9.6E-07) (p-values compared to matched LED-RL group that was not treated with resveratrol). Error bars represent standard error of the mean. (* represents P < .05).

High fluence LED-RL increased intracellular fibroblast ROS. LED-RL at fluences of 480, 640, and 800 J/cm^2^ significantly increased ROS levels in a dose-dependent manner as measured by DHR (Fig 1A and 1B). These increases in ROS were prevented by pretreating cells with 0.0001% or 0.001% resveratrol. ROS levels are presented as median fluorescent intensity of DHR of treated cells relative to matched controls. ROS generation of 480, 640, and 800 J/cm^2^ LED-RL was 132.8±2.5% (p = 0.0006), 151.0±5.2% (p = 0.0001), 158.4±1.3% (p = 0.0018), respectively, compared to 100.0±2.2% in control. Pretreatment with 0.0001% resveratrol significantly reduced ROS generation of 480, 640, and 800 J/cm^2^ LED-RL to 127.6±1.3 (p = 0.0005), 132.8±2.1 (p = 0.0048), and 131.2±5.1 (p = 0.0002), respectively (p-values compared to matched LED-RL group that was not treated with resveratrol). Pretreatment with 0.001% resveratrol significantly reduced ROS generation of 480, 640, and 800 J/cm^2^ LED-RL to 107.5±1.4 (p = 2.3E-05), 116.2±1.7 (p = 0.0002), and 117.8±1.9 (p = 9.6E-07) (p-values compared to matched LED-RL group that was not treated with resveratrol).

**Fig 2:**
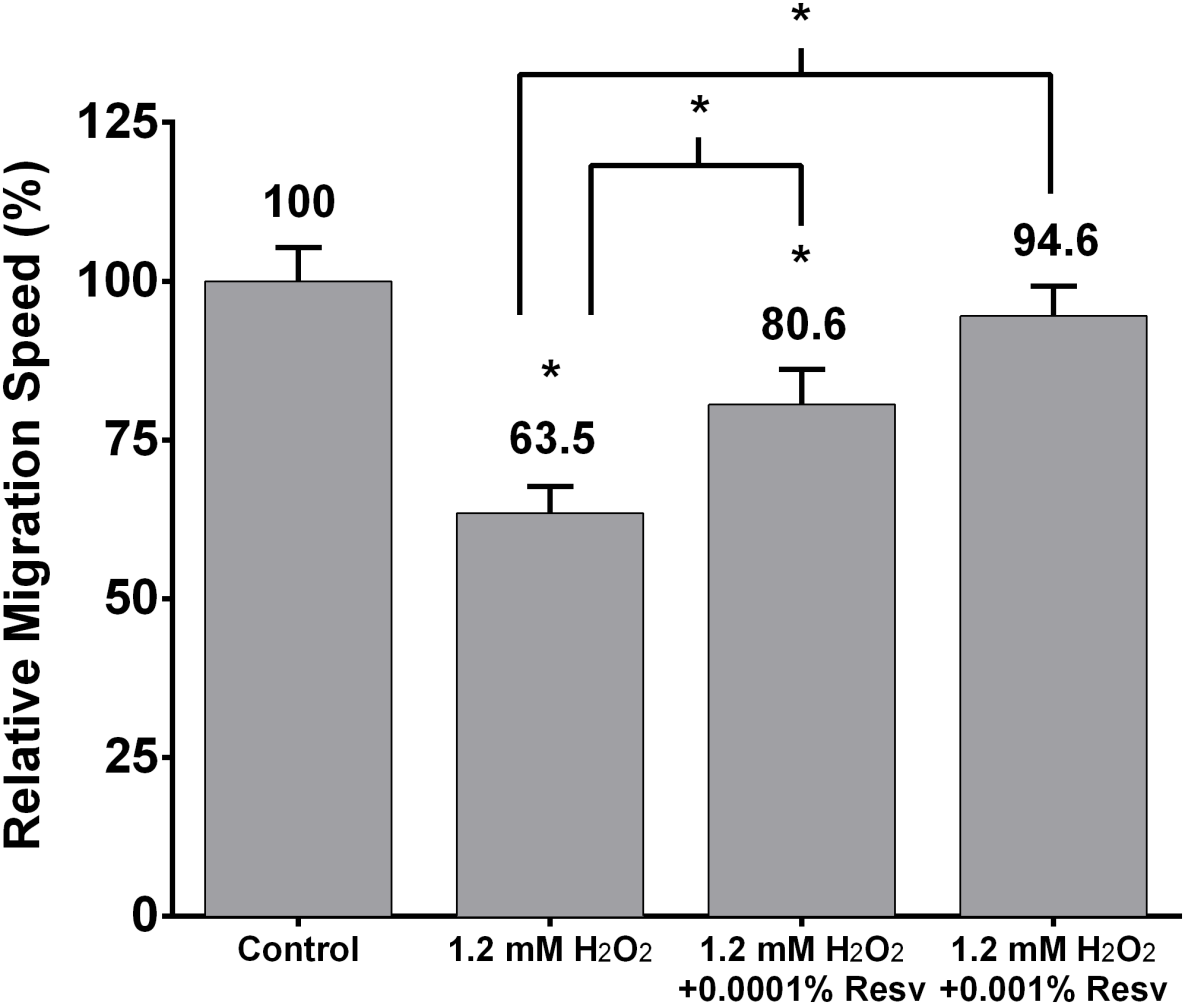
Pretreatment of fibroblasts with 1.2 mM H_2_O_2_ for 30 minutes significantly decreased relative fibroblast migration speed to 63.5±4.2% (p = 4.28–07) compared to matched controls (100±5.3%). Pretreatment with 0.0001% resveratrol significantly prevented the effects of H_2_O_2_ (1.2 mM) compared to LED-RL alone and resulted in a relative migration speed of 80.6±5.5% (p = 0.024). Pretreatment with 0.001% resveratrol significantly prevented the effects of H_2_O_2_ (1.2 mM) compared to LED-RL alone and resulted in a relative migration speed of 94.5±4.7% (p = 0.001). Error bars represent standard error of the mean. (* represents P < .05).

We next assessed the effects of ROS generated by 1.2 mM H_2_O_2_ on fibroblast migration speed and the ability of 0.0001% and 0.001% resveratrol to prevent ROS-associated changes in fibroblast migration speed. Pretreatment of fibroblasts with 1.2 mM H_2_O_2_ for 30 minutes significantly decreased relative fibroblast migration speed to 63.5±4.2% (p = 4.28–07) compared to matched controls (100±5.3%) (Fig 2). Pretreatment with 0.0001% resveratrol prevented the effects of H_2_O_2_ (1.2 mM) compared to LED-RL alone and resulted in a relative migration speed of 80.6±5.5% (p = 0.024). Pretreatment with 0.001% resveratrol significantly prevented the effects of H_2_O_2_ (1.2 mM) compared to LED-RL alone and resulted in a relative migration speed of 94.5±4.7% (p = 0.001).

**Fig 3:**
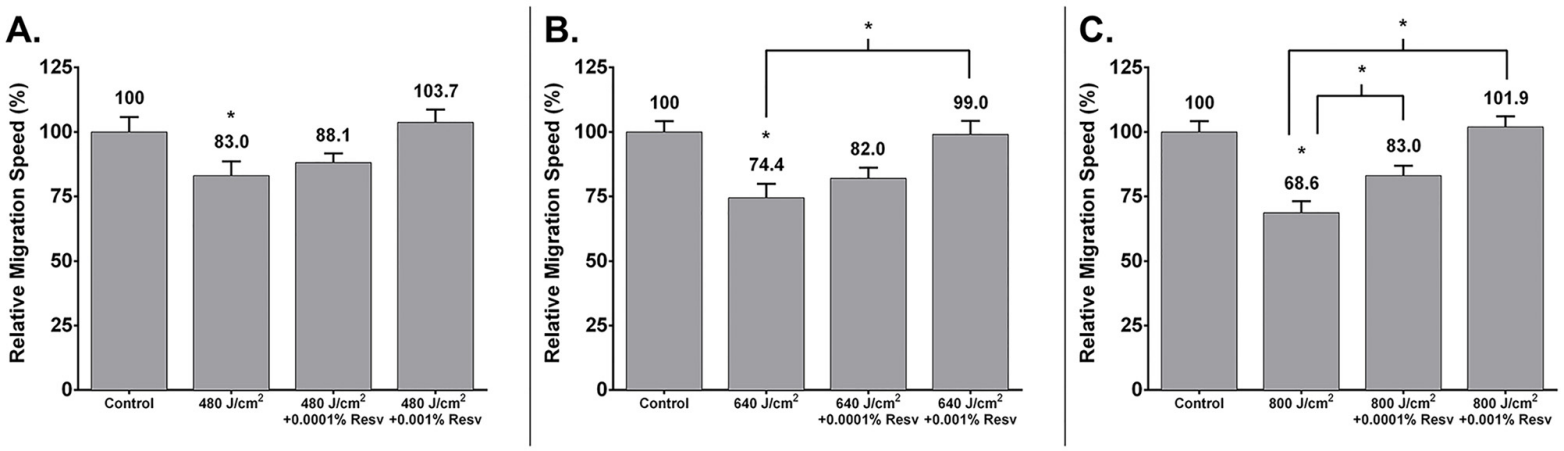
Light-emitting diode generated red light (LED-RL) at a fluence of 480 J/cm^2^ significantly decreased relative fibroblast migration speed to 83.0±5.6% (p = 0.0393) compared to matched controls (100±5.8%). **A.** Pretreatment with 0.0001% resveratrol partially prevented LED-RL’s (480 J/cm^2^) effects compared to LED-RL alone and resulted in a relative migration speed of 88.1±3.5% (p = 0.4837). Pretreatment with 0.001% resveratrol partially prevented LED-RL’s (480 J/cm^2^) effects compared to LED-RL alone and resulted in a relative migration speed of 103.7±5.0% (p = 0.0623). Error bars represent standard error of the mean. (* represents P < .05). Fig 3B. Light-emitting diode generated red light (LED-RL) at a fluence of 640 J/cm^2^ significantly decreased relative fibroblast migration speed to 74.4±5.5% (p = 0.0003) compared to matched controls (100±4.2%). Pretreatment with 0.0001% resveratrol partially prevented LED-RL’s (640 J/cm^2^) effects compared to LED-RL alone and resulted in a relative migration speed of 82.0±4.1% (p = 0.3068). Pretreatment with 0.001% resveratrol significantly prevented LED-RL’s (640 J/cm^2^) effects compared to LED-RL alone and resulted in a relative migration speed of 99.0±5.3% (p = 0.0009). Error bars represent standard error of the mean. (* represents P < .05). Fig 3C. Light-emitting diode generated red light (LED-RL) at a fluence of 800 J/cm^2^ significantly decreased relative fibroblast migration speed to 68.6±4.5% (p = 0.0003) compared to matched controls (100±4.2%). Pretreatment with 0.0001% resveratrol significantly prevented LED-RL’s (800 J/cm^2^) effects compared to LED-RL alone and resulted in a relative migration speed of 83.0±3.9% (p = 0.019). Pretreatment with 0.001% resveratrol significantly prevented LED-RL’s (800 J/cm^2^) effects compared to LED-RL alone and resulted in a relative migration speed of 101.9±4.2% (p = 0.0006). Error bars represent standard error of the mean. (* represents P < .05).

High fluence LED-RL decreased fibroblast cell migration speed. LED-RL at a fluence of 480 J/cm^2^ significantly decreased relative fibroblast migration speed to 83.0±5.6% (p = 0.0393) compared to matched control (100±5.8%) (Fig 3A). Pretreatment with 0.0001% and 0.001% resveratrol prevented LED-RL (480 J/cm^2^) effects compared to LED-RL alone and resulted in relative migration speeds of 88.1±3.5% (p = 0.4837) and 103.7±5.0% (p = 0.0623), respectively. LED-RL at a fluence of 640 J/cm^2^ significantly decreased relative fibroblast migration speed to 74.4±5.5% (p = 0.0003) compared to matched control (100±4.2%) (Fig 3B). Pretreatment with 0.0001% and 0.001% resveratrol prevented LED-RL (640 J/cm^2^) effects compared to LED-RL alone and resulted in relative migration speeds of 82.0±4.1% (p = 0.3068) and 99.0±5.3% (p = 0.0009), respectively. LED-RL at a fluence of 800 J/cm^2^ significantly decreased relative fibroblast migration speed to 68.6±4.5% (p = 0.0003) compared to matched control (100±4.2%) (Fig 3C). Pretreatment with 0.0001% or 0.001% resveratrol significantly prevented LED-RL’s (800 J/cm^2^) effects compared to LED-RL alone and resulted in relative migration speeds of 83.0±3.9% (p = 0.019) and 101.9±4.2% (p = 0.0006), respectively.

## Discussion

High fluence LED-RL decreases skin fibroblast migration speed. Mechanistically, the high fluence LED-RL decrease in migration speed is associated with a dose-dependent increase in intracellular ROS. We previously have demonstrated that resveratrol has antioxidant properties in skin fibroblasts. To investigate the role of high fluence LED-RL mediated ROS, we used resveratrol at previously studied doses that demonstrated antioxidant effects to confirm that prevention of intracellular ROS generated by LED-RL prevented the concomitant inhibition of migration.

Our study results demonstrate that LED-RL at fluences of 480, 640, and 800 J/cm^2^ increase ROS in a dose dependent manner and decrease fibroblast migration speed. These LED-RL associated increases in ROS were prevented by pretreating cells with resveratrol. We next demonstrated that H_2_O_2_-associated ROS inhibition of fibroblast migration speed could be reversed by resveratrol. Furthermore, we found that LED-RL at 480, 640 and 800 J/cm^2^ decreased fibroblast migration speed. We hypothesized that these dose-dependent decreases in fibroblast migration speed were due to increases in ROS generation and used hydrogen peroxide to confirm the role of increased ROS in decreased migration speed. Pretreatment with 0.0001% resveratrol partially prevented the LED-RL associated decrease in migration speed, while pretreatment with 0.001% resveratrol completely prevented the LED-RL associated decreases in migration speed.

To our knowledge, this is the first report that resveratrol is capable of preventing the effects of high-fluence visible red light on human skin fibroblast ROS generation and migration speed. Previous research indicates that fibroblast migration speed may play a key role in the pathogenesis of skin fibrosis and may underlie one of the underappreciated therapeutic challenges associated with skin fibrosis.[3,4] Our findings are important as they provide a mechanistic understanding of how high fluence LED-RL may induce photoinhibitory effects on human skin fibroblasts via increases in ROS generation. Elucidating the mechanisms that underlie visible light phototherapy could assist in optimizing anti-fibrotic LED-RL phototherapy settings and may provide the basis for development of additional antifibrotic adjunctive therapies. Furthermore, understanding these LED-RL associated mechanisms is important as it may reveal potential therapeutic targets for treating conditions associated with pathologic fibroblast states that clinically feature skin fibrosis.

A limitation of this study is that we used cultured human skin fibroblasts. Cultured fibroblasts do not completely recapitulate the complex physiologic state of fibroblasts that are contributing to pathologic states leading to skin fibrosis in vivo. However, use of cultured cells may also be considered a strength of this study since we used fibroblasts available from a commercial cell repository. Therefore, these results can be repeated and confirmed by other investigators. Another limitation of our study is the inability to ascribe resveratrol effects on fibroblast migration speed and ROS production as purely a function its antioxidant properties, as resveratrol may function through other cellular pathways. We believe our use of commercially available LEDs as a visible light source is a strength of our study as other researchers may utilize these arrays for photobiomodulation research.[7,25]

## Conclusion

High fluence LED-RL increases ROS and is associated with decreased fibroblast migration speed. We provide mechanistic support that decreased migration speed is associated with high fluence LED-RL generated ROS through the use of resveratrol to prevent high fluence LED-RL associated migration speed change. These data lend support to an increasing scientific body of evidence that high fluence LED-RL has anti-fibrotic properties. We hypothesize our findings may result in a greater understanding of the fundamental mechanisms underlying visible light interaction with skin and we anticipate clinicians and other researchers may utilize these pathways for patient benefit.
